# Feed Forward Artificial Neural Network: Tool for Early Detection of Ovarian Cancer

**DOI:** 10.3797/scipharm.1105-11

**Published:** 2011-07-05

**Authors:** Ankita Thakur, Vijay Mishra, Sunil K. Jain

**Affiliations:** 1 Department of Biosciences and Bioengineering, Indian Institute of Technology Bombay, Powai, 400076, MH, India; 2 Department of Pharmaceutics, Adina Institute of Pharmaceutical Sciences, Sagar, 470002, M.P., India

**Keywords:** Ovarian cancer, Neural networks, SELDI, Serum proteomics

## Abstract

Pathological changes in an organ or tissue may be reflected in proteomic patterns in serum. The early detection of cancer is crucial for successful treatment. Some cancers affect the concentration of certain molecules in the blood, which allows early diagnosis by analyzing the blood mass spectrum. It is possible that exclusive serum proteomic patterns could be used to differentiate cancer samples from non-cancer ones. Several techniques have been developed for the analysis of mass-spectrum curve, and use them for the detection of prostate, ovarian, breast, bladder, pancreatic, kidney, liver, and colon cancers. In present study, we applied data mining to the diagnosis of ovarian cancer and identified the most informative points of the mass-spectrum curve, then used student t-test and neural networks to determine the differences between the curves of cancer patients and healthy people. Two serum SELDI MS data sets were used in this research to identify serum proteomic patterns that distinguish the serum of ovarian cancer cases from non-cancer controls. Statistical testing and genetic algorithm-based methods are used for feature selection respectively. The results showed that (1) data mining techniques can be successfully applied to ovarian cancer detection with a reasonably high performance; (2) the discriminatory features (proteomic patterns) can be very different from one selection method to another.

## Introduction

Ovarian carcinoma has the highest mortality rate and form the leading cause of death from gynaecologic malignancy, which is due to its late initial diagnosis in addition to recurrence of ovarian cancer associated with resistance to therapy [1–3] resulting in approximately 21,880 estimated new cases in 2010 with an estimated 13,850 deaths in 2010 in the USA [4]. Among ovarian malignancies, approximately 90% epithelial ovarian cancer is arising from the ovarian surface epithelium (OSE) [5]. Because of late diagnosis, less than 20% of stage IV and less than 40% of stage III patients with ovarian cancer survive for 5 years [6]. The failure of early diagnosis includes mistaking symptoms for a gastrointestinal disease and unavailability of adequate sensitive screening and detection methods for precise detection of ovarian cancer in its primitive stage.

A conventional methodology for early stage detection of ovarian cancer includes application of biomarkers like CA125, but this strategy is restricted by its high cost and labour intensity [7]. Another approach, cDNA microarrays, has also been used to recognize unregulated genes in cancer tissue, using prostasin, osteopontin, and He4 markers [8–10]. A limitation of the cDNA microarray approach is that transcriptional differences in the tumor do not completely emulate the protein observed peripherally, because change in protein patterns found in blood is due to protein–protein interactions and post-translational modifications.

Recently, a protein chip tied with Surface Enhanced Laser Desorption/Ionization Time-Of-Flight Mass Spectrometry (SELDI-TOF MS) has been developed to assist protein profiling of complex biological mixtures, with high efficacy of discovering the cancer protein markers in serum or plasma [11].

Combinations of SELDI-TOF MS with bioinformatics approach successfully establish some new biomarkers and achieved high sensitivity and specificity for ovarian cancer diagnosis [11–13]. Recently the application of feature extraction techniques to proteomic data is widely used for cancer detection. [14–17]. Sorace and Zhan [18] used mass spectrometry serum profiles to detect early ovarian cancer while Petricoin *et al.* [11] applied genetic algorithm in combination with self-organizing cluster analysis for identifying ovarian cancer and reported a discriminatory pattern for ovarian cancer, which was defined by the amplitudes at 5 key M/Z values. This technique gave a sensitivity of 100% and a specificity of 95%.

The mass spectrum data present a curve with peaks and valleys, where the x-coordinate is the ratio of molecular weight to the net electrical charge for a specific organic molecule, with Dalton (Da) as unit, and biomarker identification. Although proteomic mass spectra has shown the promising potential of finding disease-related protein patterns but still some challenges remained unsolved.

The objective of this study is to determine whether SELDI-TOF MS profiling of plasma proteins coupled with an artificial intelligence data analysis algorithm could efficiently distinguish between normal controls and patients with malignant ovarian cancer. Using a standardized training set, we demonstrated that our SELDI protein profiling approach could accurately distinguish between plasma from patients with ovarian cancer and that from women having no ovarian disease.

In the present study, an alternative approach of feature selection from mass spectroscopy data of ovarian cancer is proposed. This article introduces the method of serum protein analysis based on feature selection using student t-test and classification with neural networks, establishing a new pattern for diagnosis of ovarian cancer.

## Results and Discussion

Ovarian cancer lacks any apparent early detection or screening test, which means that most cases remain undiagnosed until they have reached advanced stages. About 90% of patients are diagnosed at stage III or stage IV cancer, when it has already spread beyond the ovaries and for these patients 5 year survival rates are less than 30%. However, the small percentage of patients diagnosed with stage I ovarian cancer confined to the ovaries have a 5 year survival rate in excess of 90%.

The exact cause of ovarian cancer is unknown, which hampers the focus on early detection of ovarian cancer. The connection between diagnosis and survival in ovarian cancer has provided a rationale for efforts to improve results obtained by early stage detection. However, it is not confirmed whether the currently available screening methods can detect ovarian cancer sufficiently early to permit interference to change the normal record of the disease. There are no standard recommendations for screening for ovarian cancer. Screening women with pelvic ultrasound or blood tests, such as the evaluation of the tumor marker CA 125 has not been found to be very effective and is not recommended.

### Ovarian cancer detection

In present work pre-processing of mass spectra is done by selecting the most significant features that confine the majority of the biological information unseen in the original noisy spectra using student t-test and at last performing high-accuracy classification using feed forward neural network to spectra (Scheme 1). A special class of ANN i.e. Multi layer Perception (MLP) is applied. In MLP, the neurons are structured into layers and there are no lateral connections between neurons in the same layer and no feedback connections to neurons in previous layers. Thus, such networks are also named as Feed-Forward Artificial Neural Networks (FFANNs). In MLP, the biased sum of the inputs and bias terms are passed to activation level through a transfer function to produce the output, and the units are arranged in a layered feed-forward topology. The hidden layer enormously increases the learning power of the MLP. For MLP network architecture, a single hidden layer with sigmoid activation function, which is optimal for the dichotomous outcome, is chosen. The transfer function is selected such that the algorithm requires a response function with a continuous, single-valued with first derivative existence. Further, the transfer or activation function of the network modifies the input to give a required output.

**Fig. 1.**
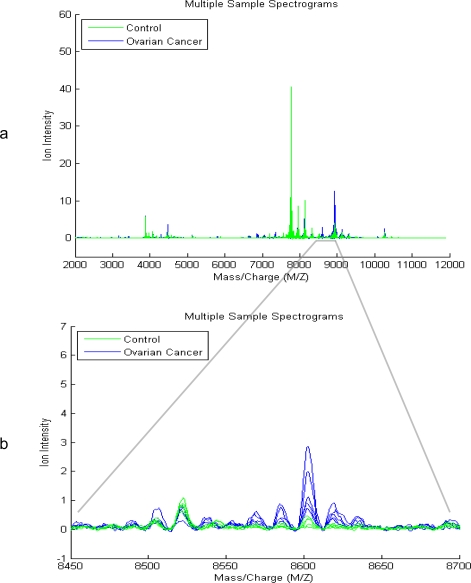
a: This study uses the high-resolution Ovarian Cancer data set that was generated using the WCX2 protein array. The sample set includes 121 cancer, 95 normal state. Plot of some data sets into a Figure window to visually compare profiles from the groups; in these graph 5 spectrograms from Ovarian Cancer patients (blue) and 5 from control patients (green). b: Zooming in on the region from 8450 to 8700 M/Z shows some peaks that might be useful for classifying the data.

**Fig. 2.**
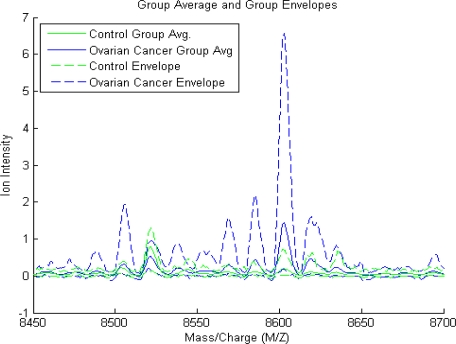
Plot of the group average and the envelopes of each group. Observe that apparently there is no single feature that can discriminate both groups perfectly.

**Fig. 3.**
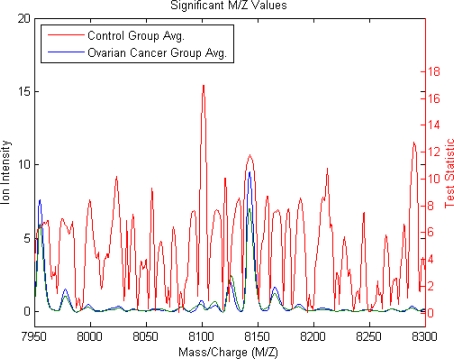
Plot of ranked features. Note that there are significant regions at high M/Z values but low intensity (∼8150 Da). The approaches to measure class separability are performed using in ranking features, such as student t-test.

Comparison of mass spectra from two initial datasets which include 95 controls (green) and 121 ovarian cancer patients (blue) is made (Fig. 1a). The small subset (5 from both) along the spectrum x-axis is identified and evaluated as significant because the prototype of amplitudes at these M/Z values entirely split out the serum of ovarian cancer patients from the controls. The finest discriminatory pattern in N-space for ovarian cancer is given by the amplitudes at the input M/Z values between 8450–8740 which can be visualized in the magnified view of the spectrum (Fig. 1b). However, the plot of the group average and the envelopes of each group show no significant feature for absolute discrimination between ovarian cancer patient and controls (Fig. 2). Therefore, to determine class discrimination, we implemented ranking the features approach using student t-test. Fig. 3 represents the plot of ranked features. It can be observed that there are significant regions at high M/Z values but have low intensity (∼8150 Da). This peak is visibly clear from the background noise.

### Overall performance of proposed method

The results for estimated testing accuracy and training accuracy of this classifier have been shown in Table 1. This end result gave 100% training accuracy in active and controlled state ovarian cancer patient with 99.16% and 98.50% testing accuracy in active and controlled state ovarian cancer patient respectively. Training, eliminate inappropriate and/or redundant data points (features) from the data (feature) set, and discovers the smallest size subset of data points as features that holds sufficient information to execute a well-organized pattern of classification.

### Comparison with the Linear Discriminant Analysis (LDA) classifier method

Linear Discriminant Analysis (LDA) is a non-parametric method [19] that is also a special form of a maximum probability discriminant rule for multivariate normal class densities with the same covariance matrix. In LDA method the number of features that can be handled has to be smaller than the number of observations. For that reason, we cannot use all the intensity values from an MS data set for these classification methods. As an alternative, we have to recognize certain M/Z ratios as inputs to these methods, and it is obvious that this feature selection step is crucial in the analysis of MS data and comparison of various methods. To construct the evaluation as suitable as possible, we feed the similar set of M/Z ratios to LDA classification method and compare its performance on our data. It is important to note that the intensities of the selected M/Z features are of particularly small amplitude, indicating at finest only 2% of the intensity of the most abundant peaks present in the serum mass spectra.

In present study we have also compared results acquired with LDA classification method to distinguish ovarian cancer patients from normal individuals based on MS data obtained by serum samples. Table 2 shows the result for the integrated feed forward ANNs classifier, the estimated overall sensitivity was found to be 98% and the estimated positive predictive value was found to be 96%. The same data were used by linear discriminant analysis, in original grouped data; the estimated overall sensitivity was found to be 85%, and the estimated specificity was 71%.

On comparison to LDA methods, our method has the benefit of not involving the number of variables used to be less than the number of subjects in the study, which is an obvious advantage for the study of MS data as the number of M/Z versus intensity data points is very large. The result of major discrimination among cancer and control groups at high M/Z values signifies that concentration should be focused in this particular region (8450–8740) only (Fig. 1a and 1b, 2). Particularly, after excluding the uncertain section and noise effects this region can be establish to propose the best for ovarian cancer diagnostic test development. The whole data set can be visualized by looking at average signal for the control and cancer samples which can be observed by the plot of the group average and the envelopes of each group (Fig. 2). Significant features can be determined by assuming that each M/Z ratio is independent. Rank features return an index to the most significant M/Z values, by the absolute value of the test statistic. Comparisons (t-testing) revealed that the variation in the mass spectra between ovarian cancer patient and controls was statistically distinguishable from the variance within the method itself, as indicated by plot of ranked features (Fig. 3).

In this paper, we presented a learning method to examine ovarian cancer proteomics data that utilized ANN as a classifier and feature selection scheme in a cross validation framework. The accuracy of tools depend on several factors such as size, quality of training set, and also the parameters chosen to represent input. In this preliminary evaluation, only two data sets from ovarian cancer database are used. The chosen feature sets combined with the neural network can provide a good solution for automatic ovarian cancer diagnosis system in the future.

## Experimental

### Materials

Present research is based on the serum proteomic analysis of the input Serum SELDI spectra data from patients with ovarian cancer and a healthy screening population. Serum MS data set is used to recognize serum proteomic patterns to discriminate the serum of ovarian cancer patients from healthy controls. The dataset was downloaded from the FDA-NCI Clinical Proteomics Program Databank [20]. This study uses the high-resolution ovarian cancer data set generated by WCX2 protein array. The sample set includes 95 controls and 121 ovarian cancer.

### Methods

#### Feature Selection

For the feature selection, every point of mass spectrum curve was observed as a feature and the equivalent ion intensity as its value. The significant features were selected by the calculation of mean intensity values for every point in mass spectra of cancer and control groups. Student t-test was employed as a method to separate both the groups. Two-sample student t test considered each feature independently. It was assumed that both groups of data values were distributed normally and had similar variances. Test statistics were calculated as follow:

$$t=\frac{X_{d}\;-\;X_{c}}{\sqrt{\left(\frac{{\text{Var}}_{d}}{n_{d}}+\frac{{\text{Var}}_{c}}{n_{c}}\right)}}$$

Where,

X_d_ and X_c_: mean values of intensities from disease and control group.
Var_d_ and Var_c_: variances of two distributions.
n_d_ and n_c_: numbers of instance in each distribution.

This t value followed student t distribution with (n_d_ + n_c_ − 2) degree of freedom. On the basis of test statistics and t distribution, the significance p value was calculated. The main features for classification are shown in Fig. 3.

#### Artificial Neural Network

Artificial Neural Networks (ANNs) are computer-based algorithms that are modelled on the organization and performance of neurons in the human brain and can be trained to identify and classify complex patterns. Pattern recognition is accomplished by regulating parameters of the ANN by a method of error reduction through learning from training. It can be standardized by applying any kind of input data, such as proteomics data generated by SELDI-TOF MS, and the output can be grouped into any given number of categories.

In present work, neural network classifier was designed, trained and tested using the feature sets described above. In training, data from different files of ovarian cancer mass-spectrometry database were selected as representatives of various classes. The classifier was tested over a large set of database for robustness. This segment describes the methods used in design, training, and testing the neural network classifier.

#### Design

A Feed-Forward Multi-Layer Perception (FFMLP) neural network with a single hidden layer is implemented for classification [21]. It is the most widely used neural network method for pattern recognition [22, 23]. Multi-Layer Perception is composed of three layers i.e. an input layer, an output layer, and one or more hidden layers, that take out useful information during learning and give modifiable weighting coefficients to components of the input layers. We explored various other implementations empirically selected this particular network since it achieved the best performances. The number of neurons in the hidden layer was set equal to five neurons. All computations were performed in Matlab® version R2008a (The Mathworks, Natick, MA, USA).

#### Training and Testing

During training, MLPs fabricate a multidimensional space, defined by the start of the hidden nodes, so that patients as well as controls are as distinguishable as possible. In the present study, total 216 files of ovarian cancer were taken which formed 1 Test set and include 173 (which is 80% of total files taken) training subset and 43 (20%) test subset. These files are selected as representatives of Active and Controlled state. The selected two classes are categorised using one against all.

#### Classifier Performance Measures

We quantified our classifier performance using the most common metrics found in literature: accuracy, sensitivity, and Specificity.

Accuracy:

It is the most crucial metric for determining overall system performance and is the test’s total accurate diagnosis of the sick and the healthy. The overall accuracy (A) of the classifier for each file is as follows [22]:

$$\text{A}\;=\;100\;\left(1\;-\;\frac{N_{e}}{N_{b}}\right)$$

The variables N_e_ and N_b_ represent the total number of classification errors and beats in the file, respectively.

Sensitivity:

It is the ability to distinguish the sick from the true ill [24]. It measures how effectively a classifier recognizes data of a certain class without missing them.

$$S_{e}\;=\;\left(\frac{T_{p}}{F_{p}\;+\;F_{N}}\right)$$

Where T_p_, F_p_, and F_N_ denote true positives, false positives and false negatives respectively.

Specificity:

It is the probability that a person who does not have a disease will be correctly identified by a clinical test. Specificity measures how exclusively it classifies beats of a certain type.

True positives are proteomic data segment which have been precisely assigned to a certain class whereas false positives are data which have been incorrectly assigned to that same class. False negative is denoted as a result that appears negative but fails to reveal a situation. A test result that shows no evidence of the disease or abnormality being investigated although the condition is actually present.

#### Analytical Procedure

The serum mass spectrum data is used as an input for analysis. Each mass spectrum data is composed of 12,000 M/Z values on the x-axis with corresponding intensities of ovarian cancer patients and controls on the y axis. The output of the algorithm is the robust separation of amplitudes at defined M/Z values that finest isolates the initial data.

The t-statistics is applied to pre-select a set of variables as inputs for classifiers for which variables are ranked, *i.e.* M/Z ratios, based on normalized difference between two groups (cancer and control groups), and then the variables are chosen on the basis of the absolute values of t-statistics.

All candidate subset includes 5 of the 12000 potential x-axis values that depict the spectra and the robustness of the test consists of plotting the pattern generated by the collective y-axis amplitudes of the candidate set of key M/Z values in N-dimensional space, where N is the number of M/Z values in the test set. The pattern produced by the virtual amplitude of the spectrum data for this set of selected values is charged for its capability to differentiate the two preliminary populations. The M/Z values within the highest rated sets are reshuffled to form new subset candidates and the resultant y-axis-defined amplitudes are rated iteratively until the set that fully discriminates the preliminary set emerges. All computations used in this study are performed in Matlab® version R2008a (The Mathworks, Natick, MA, USA).

## Authors’ Statement

### Competing Interests

The authors declare no conflict of interest.

## Figures and Tables

**Sch. 1.**
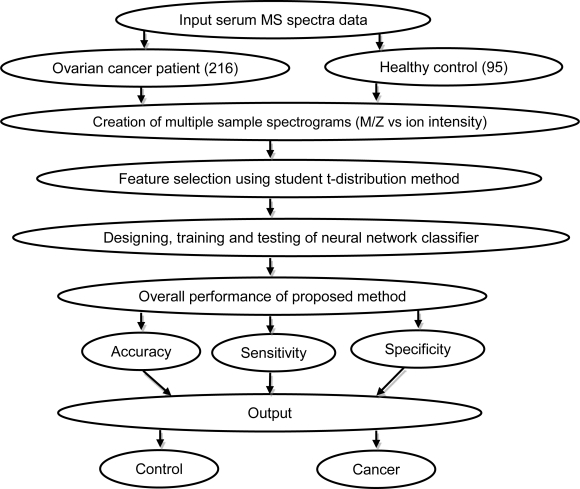
Algorithmic approach used in present study

**Tab. 1.** The Overall performance of the proposed method

**Type of Data**	**Number of Dataset used for Training**	**Number of Dataset used for Testing**	**% Training Accuracy**	**% Testing Accuracy**
Active Ovarian Cancer	100	11	100	99.16
Controlled Ovarian Cancer	80	95	100	98.50

**Tab. 2.** The comparison with the LDA classifier method

**Classifier**	**Overall Sensitivity**	**Specificity**
LDA	85%	71%
Feed Forward ANN	98%	96%
